# Long-term outcomes of ADEM-like and tumefactive presentations of CNS demyelination: a case-comparison analysis

**DOI:** 10.1007/s00415-024-12349-6

**Published:** 2024-06-11

**Authors:** Simon V. Arnett, Kerri Prain, Sudarshini Ramanathan, Sandeep Bhuta, Fabienne Brilot, Simon A. Broadley

**Affiliations:** 1 School of Medicine, Menzies Health Institute Queensland, Gold Coast Campus, Griffith University, Gold Coast, QLD 4222 Australia; 2 Department of Neurology, Gold Coast University Hospital, Southport, QLD 4215 Australia; 3 Department of Immunology, Pathology Queensland, Royal Brisbane and Women’s Hospital, Herston, QLD 4006 Australia; 4 Neuroimmunology Group, Kids Neurosciences Centre, Faculty of Medicine and Health, Children’s Hospital at Westmead, University of Sydney, Westmead, NSW 2145 Australia; 5 Department of Neurology, Concord Hospital, Sydney, NSW 2139 Australia; 6 Griffith university, Gold Coast Campus, Gold Coast, Queensland Australia

**Keywords:** Multiple sclerosis, Tumefactive, Acute disseminated encephalomyelitis, Prognosis

## Abstract

A minority of initial multiple sclerosis (MS) presentations clinically or radiologically resemble other central nervous system (CNS) pathologies, acute disseminated encephalomyelitis (ADEM) or tumefactive demyelination (atypical demyelination presentations). With the aim of better defining the long-term outcomes of this group we have performed a retrospective cohort comparison of atypical demyelination versus ‘typical’ MS presentations. Twenty-seven cases with atypical presentations (both first and subsequent demyelinating events) were identified and compared with typical MS cases. Disease features analysed included relapse rates, disability severity, whole brain and lesion volumes, lesion number and distribution. Atypical cases represented 3.9% of all MS cases. There was considerable overlap in the magnetic resonance imaging (MRI) features of ADEM-like and tumefactive demyelination cases. ADEM-like cases tended to be younger but not significantly so. Atypical cases showed a trend towards higher peak expanded disability severity score (EDSS) score at the time of their atypical presentation. Motor, cranial nerve, cerebellar, cerebral and multifocal presentations were all more common in atypical cases, and less likely to present with optic neuritis. Cerebrospinal fluid (CSF) white cell counts were higher in atypical cases (*p* = 0.002). One atypical case was associated with peripheral blood myelin oligodendrocyte glycoprotein (MOG) antibodies, but subsequent clinical and radiological course was in keeping with MS. There was no difference in long-term clinical outcomes including annualised relapse rates (ARR), brain volume, lesion numbers or lesion distributions. Atypical demyelination cases were more likely to receive high potency disease modifying therapy early in the course of their illness. Despite the severity of initial illness, our cohort analysis suggests that atypical demyelination presentations do not confer a higher risk of long-term adverse outcomes.

**Supplementary Information:**

The online version contains supplementary material available at 10.1007/s00415-024-12349-6.

## Introduction

Multiple sclerosis (MS) is a chronic inflammatory disease of the CNS which often presents with recurrent episodes of focal neurological deficit in the absence of encephalopathy or fever [1, 2]. A small number of cases present with atypical clinical or radiological features suggestive of acute disseminated encephalomyelitis (ADEM) [3, 4] or cerebral neoplasia (tumefactive demyelination) [5]. The largest case series to date, retrospectively reviewing tumefactive demyelinating lesions over a period of 30 years at the Mayo Clinic, identified 183 cases meeting criteria for MS [6]. These atypical presentations are uncommon [7], pose a diagnostic dilemma, and data regarding treatment and prognosis are limited [8, 9]. Despite these difficulties, a recent review proposed that combinations of imaging and paraclinical findings can be used to diagnose tumefactive demyelinating lesions [10]

ADEM typically presents in childhood and features include altered level of consciousness, seizures, fever or focal/multifocal neurological deficits. These clinical features are accompanied by widespread, poorly demarcated predominantly white matter lesions of the same age. These features have been collated into criteria for childhood ADEM [11] however similar criteria for adult presentations are yet to be defined, and previous investigations commonly define their own criteria, leading to issues of heterogeneity in case definition. MRI of the brain and spine typically shows simultaneous multifocal demyelination throughout the brain and spine and up to 50% of cases are positive for MOG antibodies. ADEM-like presentations of MS are seen in adults but typically they do not have all of the clinical features of the childhood form and MOG antibody prevalence has been less well studied [12, 13]. Tumefactive demyelination is defined as lesions at least two centimetres in diameter and featuring gadolinium (Gd) enhancement [7–9, 14, 15]. Incomplete peripheral Gd-enhancement (‘broken ring’) is considered unique to this form of MS [8, 15]. Whilst initially described as being mono-focal, multifocal lesions feature in many series [8]. Cases with antibodies to AQP4 and MOG have been described [12, 13, 16–19]. Expert opinion and case series analysis has led to plasma exchange and immunosuppressive therapy being advocated for atypical forms of MS [13].

With the aim of further adding to knowledge of the long-term outcomes for atypical MS presentations in adults we conducted a retrospective cohort comparison study comparing clinical and radiological outcomes with age- and sex-matched typical MS cases. Our hypotheses were: (1) atypical MS cases would have worse outcomes than typical cases in terms of disability, lesion load and brain atrophy and (2) that a proportion of atypical cases would be positive for AQP4 or MOG antibodies.

## Methods

### Ethics oversight and approval

Ethics approval was sought and obtained through the Griffith University and Gold Coast Hospital and Health Service, Human Research Ethics Committees. Written informed consent was provided by all participants.

### Case ascertainment and data collection

Atypical MS presentations (both initial and subsequent) were identified through systematic review of medical records of patients under the care of the CNS inflammatory diseases clinic at the Gold Coast University Hospital. Cases were enrolled if they featured atypical clinical presenting symptoms (fever, seizure, encephalopathy, severe multifocal neurological deficits) and/or atypical MRI findings (see lesion definitions below). Typical cases matched for sex and age at onset were identified from a register of cases seen at the same clinic. We attempted to match up to three typical cases for every atypical case. Typical cases met the 2017 McDonald criteria for MS and atypical cases were also assessed against these criteria. Cases (atypical or typical) were excluded if there was insufficient data (clinical or MRI) to confirm a diagnosis of MS or provide a minimum dataset (demographics, disability, and relapse information).

The following clinical details were collected from available records and direct interview with cases: current age, sex, age at onset, relapse history, relapse frequency, time to first relapse (following initial presentation), time to expanded disability status scale (EDSS) score 6.0, final EDSS (last review), MS treatment, CSF cell counts, CSF protein, oligoclonal bands and MRI data (see below for details). Annualised relapse rate (ARR), EDSS and MRI parameters were recorded for the 2-year, 5-year and most recent clinical review available following disease onset. Clinical and MRI data were included if they were available within 6 months of each time point.

### Serological testing

Testing for AQP4 antibodies was performed by Pathology Queensland Immunology Laboratory, Brisbane using a combination of tissue-based immunofluorescence as previously described [20] and fixed cell-based assay (Eurommun®). MOG antibodies were tested by Westmead Immunology Laboratory, Sydney using a live-cell fluorescence activated cell sorting technique as previously described [20].

### Radiological lesion definitions

ADEM-like MS was defined as multiple (> 10), large (>6 mm maximum diameter in any single plane), irregularly shaped, or poorly demarcated lesions of high intensity on T2 FLAIR MRI of the brain and spine that were of the same age on DWI and Gd-enhancing sequences [21]. Tumefactive MS lesions were defined as very large (> 2 cm) lesions identified on T2 FLAIR sequences, spanning the peri-ventricular to subcortical white matter, with or without a surrounding oedema or Gd-gadolinium enhancement [21].

### MRI analysis

MRI were assessed using eFilm Workstation® 4.2.3, IBM Watson Health software on Eizo® RadiForce MX270W 68 cm monitors. MRI parameters included the number of T2 FLAIR hyperintense lesions, the neuroanatomical location of these lesions, the number of large lesions (defined as >6 mm in diameter in at least one plane), the presence and number of gadolinium-enhancing lesions, and the presence and number of T1 hypointense lesions (black holes).

The following criteria were used to determine if lesions were of the same age; No established T1 black holes (minor T1 hypointensity was permitted as can be seen in acute lesions), all large lesions (> 6 mm) showed diffusion restriction or T1 Gd-enhancement and all lesions had a poorly demarcated boarder.

Volumetric analysis was performed using the open source software 3DSlicer v4.10.2 (http://www.slicer.org) [22]. Following importation of DICOM format imaging, cranial vault and soft tissue imaging was removed using the Swiss Skull Stripper module v4.1(https://www.slicer.org/wiki/Documentation/Nightly/Modules/SwissSkullStripper, Institute for Surgical Technology and Biomechanics, University of Bern, Switzerland). Whole brain and lesion volumes were measured using the Editor module v4. 1 (https://www.slicer.org/w/index.php/Documentation/4.3/Modules/Editor, National Alliance for Medical Imaging Computing, Harvard University, US).

### Statistical analysis

Statistical comparisons between the atypical MS cohort, and whole MS database, and the age- and sex-matched typical MS cohort were performed. The first comparison used a database of person with MS (pwMS) fulfilling the 2017 revised McDonald’s criteria [23] seen at Gold Coast University Hospital over the past 17 years. These data were used to compare demographics and disease course of the atypical MS cases against an unmatched cohort. The second comparison group was an age- and sex-matched cohort of typical MS cases identified from the same database as described. Comparison of categorical data were performed using a *Χ*^2^ test and continuous data with the Kruskal–Wallis test. The effect of baseline characteristics on outcomes was assessed using forward stepwise linear regression analysis with *p* < 0.05 as the cut off for inclusion in the model. Survival analysis was undertaken using Kaplan–Meier curves and Cox proportional hazard modelling including significant predictors identified from the regression analysis of outcomes [24]. All statistical analyses were performed using the Statistical Package for Social Science (SPSS®) v25 (IBM®; Chicago, US).

## Results

### Case ascertainment

A total of 28 cases were identified on clinical or radiological grounds as meeting our criteria for atypical demyelination. One case was only ever seen once in our clinic, sometime after their atypical presentation and was excluded due to lack of clinical and imaging data. This left 27 included atypical MS cases. All these cases met the McDonald criteria for MS (excluding one case in regards to requirement for an alternative diagnosis—see below). There were 712 cases in the MS Clinic database. This gives a relative frequency of 28/712 (3.9% [95%CI 2.6–5.6%]) We identified 76 age- and sex-matched typical MS cases from the database. One of these cases was also excluded due to a lack of clinical and MRI data, leaving 75 included in the analysis.

### Atypical cases

Table 1 gives the demographic information, initial clinical features, MRI data, CSF results and antibody results for individual atypical MS cases. There were 13 ADEM-like cases and 14 tumefactive cases. We determined ADEM-like cases to be atypical demyelinating presentations rather than traditional ADEM on the basis of ADEM-like cases demonstrating combinations of CSF oligoclonal band positive status (6/8), remote MRI T1 black holes on initial MRI suggesting previous demyelinating events (8/13), presence of periventricular lesions (13/13) or subsequent relapses (6/13). Atypical presentations occurred at the onset of disease (first attack) in the majority of (23/27 (85%) cases), but a small number occurred as the second (1 case) or third attack (3 cases). When monophasic cases were excluded the number of atypical presentations occurring as first events 12/16 (75%) was higher than the expected number of 5/16 (34%) based on the mean number of relapses observed (*p* < 0.01). One case had 6 tumefactive relapses affecting both hemispheres and posterior fossa. Atypical clinical features were seen in 12/27 (44%) of atypical MS cases. Cognitive impairment at first presentation was seen in 7/13 (54%) of ADEM-like cases compared with 2/14 (14%) of tumefactive cases (*p* = 0.077). Depressed level of consciousness was seen in 4/13 (31%) of ADEM cases and none of the tumefactive cases. Two ADEM cases featured headache (7%) and one presentation involved fever in (4%). The remaining atypical MS cases (15/27 [56%]) were identified on the basis of radiological features and in some cases the symptoms were relatively mild. Lesions meeting our criteria for tumefactive demyelination were also seen in 6/13 (46%) of ADEM-like cases. The median (range) of total T2 brain lesions was greater (*p* = 0.029) for ADEM-like presentations 22 (3–85) than for tumefactive cases 2.5 (1–81). Gd-enhancement was seen in 7/11 (64%) ADEM-like and 10/12 (83%) tumefactive MS cases where contrast was administered (*p* = 0.549).

**Table 1:** Summary of clinical features from atypical MS cases

Case	Type	Demographics		Relapse	Prior	Clinical features				MRI brain T2 lesions									MRI brain T1		MRI spine T2 lesions				CSF examination			Antibodies	
		Onset age (years)	Sex	Symp episode	Infect (wks)	Cog Imp	Obtund	H/A	Focal Neurol	Tumefactive			Large (≥ 6 mm)			Small (< 6 mm)			Total	Black	Cervical		Thoracic		WCC (× 10^6^/L)	Protein (mg/dL)	OCB	AQP4	MOG
										*N*	DWI	Gd	*N*	DWI	Gd	*N*	DWI	Gd	*N*	Holes	*N*	Gd	*N*	Gd					
1	ADEM-like	24	F	1	−	+	+		+	0			19	+	−	62	+	−	81	9	0		1	−	3	340	Local	−	
2	ADEM-like	15	F	1	UTI (8)	+		+	+	3	+	+	0			56	+	+	59	26	1	+	0		10	630	Local	−	−
3	ADEM-like	51	M	1	URTI (8)	+			+	3	+	+	36	+	−	18	+	+	57	31	0		1	+	2	380	Negative	−	−
4	ADEM-like	25	F	1	−				+	0			32	+	+	15	+ / −	+	47	7	5	+ / −	3	+ / −				−	−
5	ADEM-like	16	F	1	Fever (0)	+	+		−	0			35	−	+	11	−	+	46	3	1	+	0		14	350	Local	−	−
6	ADEM-like	34	F	1	−				+	4	+	+ / −	13	+	+ / −	26	+	+ / −	43	8	0		0					−	+
7	ADEM-like	25	F	1	−				+	0			13	+	+ / −	16	+	+ / −	29	6	0		0					−	−
8	ADEM-like	17	F	1	URTI (2)	+	+	+	+	5	+	+	11	+	+	6	+	+	22	19	0		1	+	43	240	Local		−
9	ADEM-like	28	F	1	−				+	0			16	−	−	6	−	−	22	0	1							−	−
10	ADEM-like	19	F	3	−				−	2	+	−	0			18	+	−	20	0	2	−	1	−				−	−
11	ADEM-like	42	F	1	−	+			+	0			0			16	−	−	16	0					1	190	Local		−
12	ADEM-like	21	F	1	−	+			+	0			10	+ / −	−	0			10	3	3	−	4	−			Negative	−	−
13	ADEM-like	38	M	1	−				+	4	+	−	4	+	−	0			8	3	0		0		32	360	Local	−	−
14	Tumefactive	31	F	3	URTI (2)				+	1	+	+	40	+ / −	−	44	+ / −	−	85	29	1	−	3	−					
15	Tumefactive	25	F	1	−				+	1	+	+	21	+ / −	−	28	+ / −	−	50	16	1	−	0		3	200	Local	−	−
16	Tumefactive	26	M	3	−				+	1	+	+	7	+	−	1	+	−	9	0	0		0		12	330	Local	−	
17	Tumefactive	36	F	1	−				+	1	+	+	1	−	−	1	−	−	5	0	0		1	−	5	280	Local	−	
18	Tumefactive	36	M	1*		+	+		+	1	+	+	1	−	−	3	−	−	5	0					121	1800	Negative	−	
19	Tumefactive	39	F	1	−				+	1	+	+	2	−	−	1	−	−	4	0	0		0					−	−
20	Tumefactive	28	F	1	−				+	2	+	−	1	+	+	0	+	−	3	2	0		0		9	210	Local	−	−
21	Tumefactive	41	F	1	−				+	2	+	−	1	+	−	0			3	0	0		0		0	240	Negative	−	−
22	Tumefactive	28	F	1	−				+	1	+	+	1	−	−	1	−	−	3	2	0		0		10	340	Local	−	
23	Tumefactive	23	F	1	−			+	+	1	+	+	2	+	+	0			3	2	0		0		16	310	Local	−	−
24	Tumefactive	44	F	1	URTI (8)			+	+	1	+	+	0			2	−	−	3	0	1	−	0		2	210	Negative	−	−
25	Tumefactive	38	F	1	−	+			+	2	+	+	0			1	−	−	3	0	0		0			250	Systemic	−	−
26	Tumefactive	31	M	1	URTI (2)			+	+	1	+	−	1	−	−	0			2	0					3	550	Local	−	−
27	Tumefactive	31	M	1	−				+	1	−	−	1	+	−	0			2	1	1	−	3	−				−	−

*Symp Ep* symptomatic episode; *Cog Imp* cognitive impairment; *Obtund* obtundation; *Focal* focal neurological deficit; *N* number; *DWI* diffusion weighted imaging; *Gd* Gadolinium enhancement; *WCC* white cell count; *OCB* oligoclonal bands; *ADEM* acute disseminated encephalomyelitis; *F* female; *M* male; + present and/or present in all/nearly all; − absent and/or absent in all; + / − present in some lesions; *Local* locally synthesized gamma-globulin found only in CSF; *Systemic* systemically synthesized gamma-globulin found in serum and CSF

### MRI of atypical presentations

**Fig. 1:**
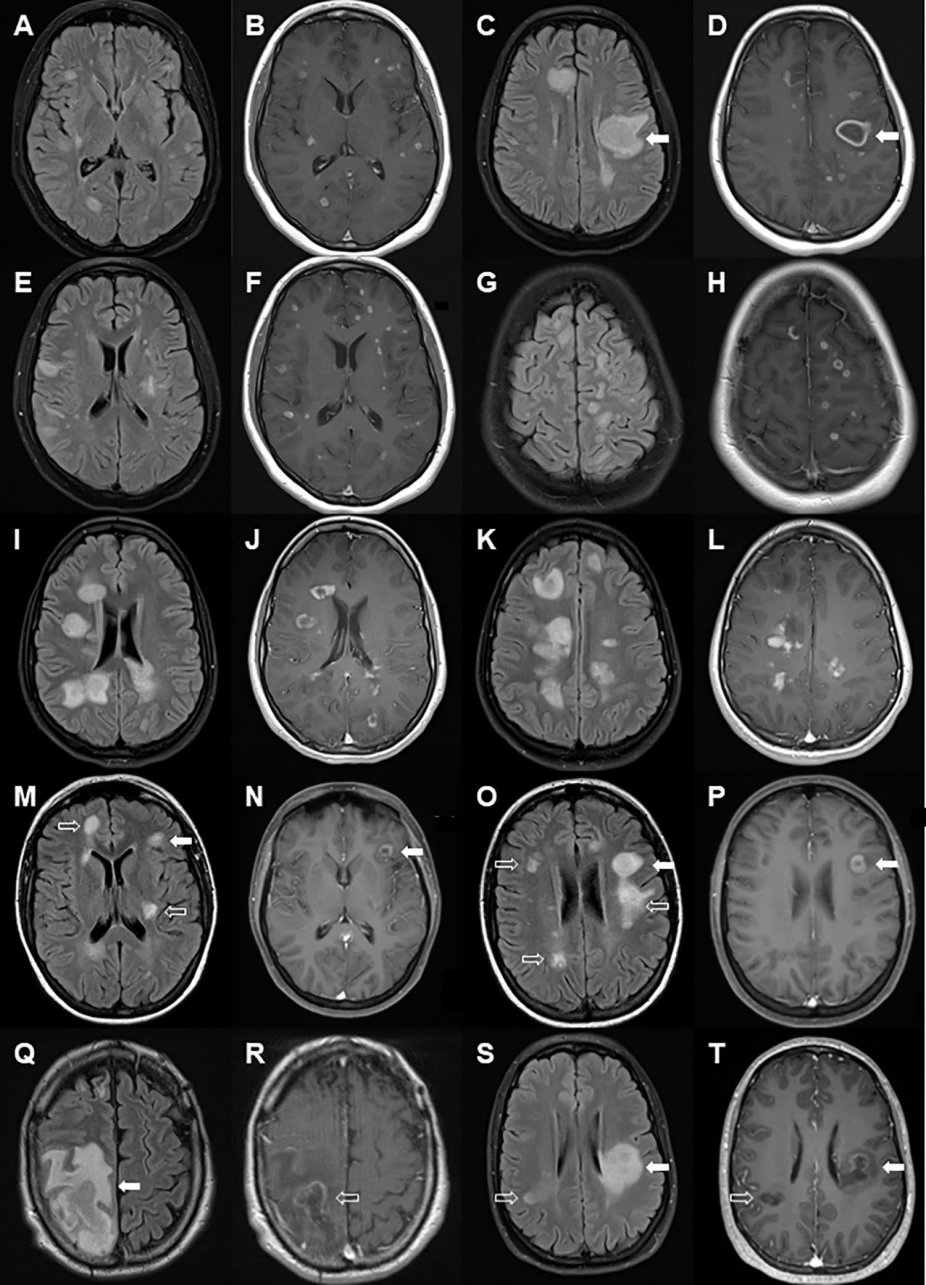
MRI of ADEM-like and tumefactive MS cases. Images are paired (matched slices) with FLAIR images in first and third vertical panels and Gd-enhanced T1 weighted sequences in second and fourth vertical panels. Case of ADEM-like lesions with small and large lesions which all show Gadolinium enhancement (**A**–**D**), one larger lesion (D arrow) shows ring-enhancement with central hypointensity and peri-lesional hypointensity with surrounding oedema. Case of ADEM-like lesions with a multitude of smaller lesions all of which show either homogeneous or ring enhancement (**E**–**H**). Case of ADEM with multiple large lesions showing both ring enhancement and heterogeneous enhancement. Case of ADEM-like lesions showing multiple small and large lesions (**M**–**N**). Some lesions are non-enhancing, with some having central hypointensity on T1 sequences (**M** and **O** open arrows) whilst other lesions show ring-enhancement (open arrows). Case of recurrent tumefactive MS showing large incomplete ring-enhancing lesion with central hypointensity and surrounding oedema (solid arrow) with mass effect (**S** and** T**). Case of tumefactive MS with a large incomplete ring-enhancing lesion (solid arrow) with central and perilesional hypointensity and a second non-enhancing lesion (open arrow) with central hypointensity (**S** and **T**)

**Fig. 2:**
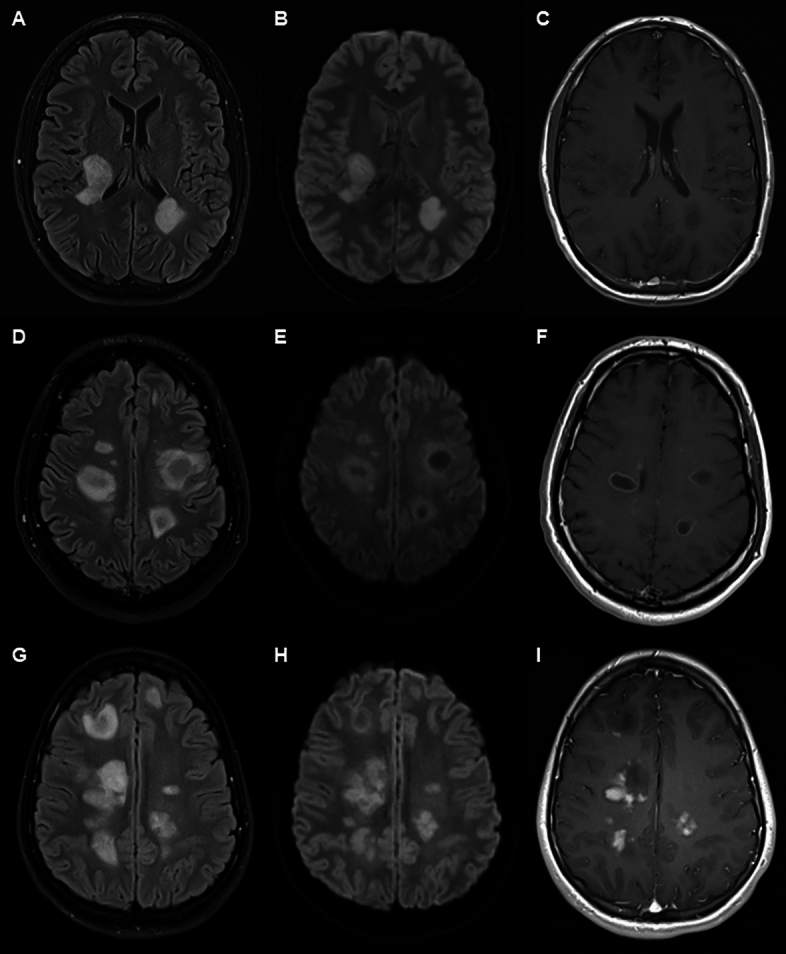
Three patterns of lesion in ADEM-like lesions. Vertical panels show FLAIR sequences (right), diffusion weighted images (centre) and T1 with contrast (left). Horizontal panels show individual cases. Upper panel (**A**–**C**) shows case with FLAIR and DWI hyperintensity with T1 hypointensity, but no Gd-enhancement. Middle panel (**D**–**F**) shows ring pattern hyperintensity on FLAIR and DWI with central hypointensity and ring-enhancement on T1 sequence. Lower panel (**G**–**I**) shows predominantly heterogeneous FLAIR and DWI hyperintensity with heterogeneous Gd-enhancement. There are additional lesions showing central T1 hypointensity and no enhancement.

Illustrative MRI features for ADEM-like and tumefactive presentations of MS are given in Fig. 1. Particular features of note included multiple enhancing lesions in ADEM-like presentations (Fig. 1B, F, J, D, H and L), peri-lesional T1 hypointensity (Fig. 1D and P), perilesional oedema (Fig. 1Q), central hypo-intensity on T1 (Fig. 1D, P, R and T), complete ring-enhancement (Fig. 1D, J and P), incomplete ring enhancement (Fig. 1R and T), homogeneous enhancement (Fig. 1l, O and S) and heterogeneous enhancement (Fig. 1K and Q). We noted three patterns of ADEM-like lesion as shown in Fig. 2 which appeared to be independent of timing of the scans in relation to onset of clinical symptoms. In the first pattern there was confluent T2 hyperintensity on FLAIR imaging matched by homogeneous hyperintensity on DWI sequences and hypodensity on T1 sequences without Gd-enhancement. In the second pattern T2 hyperintense lesions on FLAIR imaging showed central relative hypo-intensity, which was matched by similar, but more pronounced changes on DWI sequences and a clear pattern of ring-enhancement with central hypo-intensity on T1 sequences. The third pattern showed patchy central T2 hyperintensities on FLAIR imaging matched by similar changes on DWI and Gd-enhanced T1 sequences. A summary of MRI features in atypical cases is given in Supplementary Table 1.

### Comparison of baseline characteristics

Comparison of baseline characteristics between the MS database cohort, the age/sex matched typical MS cohort and the atypical MS cases, as well as between ADEM-like and tumefactive cases are shown in Table 2. There was no difference in the sex distribution of any of the groups. Whilst overall there was no difference in the age of onset between the atypical cases and MS database cases, the ADEM-like cases showed a trend towards a younger age of onset (20.5 [15–51] years) compared to tumefactive cases (29.5 [23–44] years, *p* = 0.068) and were younger than typical MS database cases (35 [14–71] years, *p* = 0.038). Peak EDSS (during the index presentation) was higher in the ADEM-like group, but this difference was not statistically significant. There was no difference in the median age of atypical and the age-matched typical MS cases. A history of recent infection was noted in 7/25 (28%) of the atypical cases and 7/54 (13%) of typical MS cases, but this difference was not statistically significant (*p* = 0.19). Atypical MS cases were more likely to have motor (*p* = 0.035), cranial nerve (*p* = 0.003), cerebellar (*p* = 0.005) and cerebral (*p* < 0.001) features in their atypical episode. They were also more likely to have multifocal attacks (*p* < 0.001), particularly in the ADEM-like group (*p* = 0.046) and were less likely to have optic neuritis (*p* = 0.037). CSF white cell count was higher in the atypical MS cases when compared to both typical MS cohorts (*p* = 0.002 for the databases and *p* = 0.052 for the matched cohort). This difference appeared to be principally driven by the tumefactive MS cases (Supplementary Fig. 1). No significant differences in CSF protein and presence of oligoclonal bands were seen. Antibodies to AQP4 were tested in 24/27 (89%) atypical cases and all were negative. MOG antibodies were tested in 21/27 (78%) of atypical cases and were positive in 1/21 (5%). This case has been treated with rituximab and MOG antibodies were negative on repeat serum testing 2 years later. MRI in this case shows features typical for MS (total of 21 white matter brain lesions, periventricular lesions, Dawson finger lesions, juxta-cortical lesions and inferior temporal lobe lesions). There had been new lesions over time, but no Gd-enhancing lesions since the ADEM-like presentation and no lesions typical for MOGAD (no lesions of the optic nerve, spinal cord, brainstem or cerebellum).

**Table 2:** Comparison of demographic and clinical features at baseline between typical and atypical MS cohorts

	Typical		Atypical			p-value		
Clinical characteristic	Database (A)	Matched (B)	All (C)	ADEM-like (D)	Tumefactive (E)	A vs C	B vs C	D vs E
*N*	712	75	27	13	14			
Sex (female)—*n*/*N* (%)	568 (80)	56 (74)	21 (78)	11 (85)	10 (71)	ns	ns	ns
Age at disease onset (years)—median (range)	35 (14–71)	33 (13–56)	28 (15–51)	20.5 (15–51)	29.5 (23–44)	ns	ns	0.068
Deficits at presentation—*n*/*N* (%)								
Sensory		29 (39)	13 (48)	7 (54)	6 (43)		ns	ns
Motor		14 (19)	11 (41)	6 (46)	5 (36)		0.035	ns
Optic neuritis		17 (23)	1 (4)	1 (8)	0 (0)		0.037	ns
Cranial nerve		11 (15)	12 (44)	7 (54)	5 (36)		0.003	ns
Incoordination		5 (7)	8 (30)	3 (23)	5 (36)		0.005	ns
Bladder/bowel dysfunction		5 (7)	2 (7)	0 (0)	2 (8)		ns	ns
Cerebral		0 (0)	9 (33)	7 (54)	2 (14)		< 0.001	0.046
Multifocal		4 (5)	15 (56)	7 (54)	8 (57)		< 0.001	ns
CSF analysis								
Protein (mg/L)—median (range)	360 (110–1700)	340 (190–1085)	320 (190–1800)	290 (190–380)	320 (210–1800)	ns	ns	ns
WCC (× 10^6^/mL)—median (range)	2 (0–390)	4 (0–390)	7 (0–121)	3 (1–43)	10 (0–121)	0.002	ns	ns
Local synthesis of oligoclonal bands—n/N (%)	168/224 (75)	28/37 (76)	13/19 (68)	6/8 (77)	7/11 (64)	ns	ns	ns
Serology—n/N (%)								
AQP4 antibody positive		0/52 (0)	0/24 (0)	0/13 (0)	0/11 (0)		ns	ns
MOG antibody positive		0/10 (0)	1/21 (5)	1/12 (8)	0/9 (0)		ns	ns

Statistical comparisons undertaken were Atypical MS vs Typical MS or Database MS and Tumefactive vs ADEM-like

*MS* multiple sclerosis; *ADEM* acute disseminated encephalomyelitis; *ns* not significant; *OCB* oligoclonal bands; *AQP4* aquaporin-4; *MOG* myelin oligodendrocyte glycoprotein

### Comparison of long-term clinical outcomes

A comparison of clinical outcomes is given in Table 3. The period of follow up for typical MS cases and consequently age at last review were higher than the atypical cases (*p* < 0.001). This affects several time dependent outcomes. In view of this we would be circumspect about the finding of a higher rate of monophasic/CIS disease in the atypical cohorts compared to both typical MS cohorts. With longer follow up this rate would be likely to fall (see time to event analysis below). Similarly, final EDSS was lower for the atypical MS cases. There was no difference in any of the disease duration standardised scores (e.g. 2 year and 5-year EDSS) and the final MSSS, which corrects for disease duration. Atypical MS cases were more likely to have subsequent motor (*p* = 0.025) and cerebral (*p* = 0.001) relapses and less likely to have optic neuritis (*p* = 0.009). Atypical cases were more likely to have been commenced on highly effective disease modifying therapy as their initial treatment compared to typical MS cases (*p* < 0.001). Subsequent escalation of treatment was conversely more common in the typical MS cohort (*p* = 0.001). This may also reflect the greater duration of follow up for the typical MS cases and a lower availability of highly effective therapies at the time of their original diagnosis.

**Table 3:** Comparison of disease outcomes in typical and atypical MS cases

	Typical MS		Atypical MS			p-value		
Clinical outcome	Database (A)	Matched (B)	All (C)	ADEM-like (D)	Tumefactive (E)	A vs C	B vs C	D vs E
*N*	712	75	27	13	14			
age at last follow up (years)—median (range)		49 (19–73)	35 (17–57)	30 (17–57)	42.5 (24–51)		< 0.001	ns
follow-up (years)—median (range)		16.0 (2.0–39.7)	5.8 (0.3–15.3)	5.8 (0.3–15.2)	6.4 (0.4–15.3)		< 0.001	ns
Clinical course—*n* (%)								
Monophasic/CIS	53 (7)	6 (8)	9 (33)	6 (46)	3 (21)	< 0.001	0.01	ns
Relapsing remitting	422 (59)	55 (73)	16 (59)	7 (54)	9 (64)			
Secondary progressive	168 (23)	10 (13)	2 (7)	0 (0)	2 (14)			
Primary progressive	69 (10)	4 (5)	0 (0)	0 (0)	0 (0)			
Time to first relapse (years)—median (95% CI)		3.0 (1.8–4.2)	2.0 (0.4–3.6)	2.0 (0.5–3.5)	2.0 (0.0–10.3)		ns	ns
Annualised relapse rate—median (range)								
To Year 2		0.0 (0.0–2.0)	0.5 (0.0–1.0)	0.0 (0.0–1.0)	0.5 (0.0–0.5)		ns	ns
To Year 5		0.2 (0.0–0.8)	0.2 (0.0–0.6)	0.2 (0.0–0.6)	0.2 (0.0–0.4)		ns	ns
Final		0.1 (0.0–0.4)	0.1 (0.0–2.4)	0.0 (0.0–0.5)	0.1 (0.0–2.4)		ns	ns
Subsequent relapse symptoms–*n* (%)								
Sensory		41/64 (64)	7/17 (41)	5/10 (50)	2/7 (29)		ns	ns
Motor		27/64 (42)	13/17 (77)	8/10 (80)	5/7 (71)		0.025	ns
Optic neuritis		28/64 (44)	1/17 (6)	1/10 (10)	0/7 (0)		0.009	ns
Brainstem		22/64 (34)	7/17 (41)	5/10 (50)	2/7 (29)		ns	ns
Cerebellar		8/64 (13)	3/17 (18)	3/10 (30)	0/7 (0)		ns	ns
Bladder/bowel dysfunction		19/64 (30)	3/17 (18)	3/10 (30)	0/7 (0)		ns	ns
Cerebral		1/64 (2)	5/17 (30)	1/10 (10)	4/7 (57)		0.001	ns
EDSS—median (range)								
Peak at presentation		2.5 (0.0–8.0)	3.0 (1.0–9.0)	3.0 (2.0–9.0)	2.75 (1.0–7.0)		ns	ns
Year 2		2.5 (0.0–7.0)	1.75 (0.0–5.0)	1.5 (0.0–5.0)	1.25 (0.0–2.0)		ns	ns
Year 5		1.5 (0.0–4.0)	1.5 (1.0–6.0)	1.25 (0.0–6.0)	1.25 (0.0–4.0)		ns	ns
Final EDSS		2.5 (0.0–7.5)	1.0 (0.0–6.5)	0.0 (0.0–2.0)	1.0 (0.0–6.5)		0.018	ns
Disability								
Time to EDSS 6.0 (years)—mean (95% CI)		24.1 (23.3–30.4)	13.5 (11.8–15.3)	13.1 (11.3–14.9)	13.1 (10.5–15.6)		ns	ns
Progression rate—median (range)		0.12 (0.00–0.97)	0.13 (0.00–3.54)	0.00 (0.00–0.93)	0.18 (0.00–3.54)		ns	ns
MSSS—median (range)		1.64 (0.03–8.49)	1.07 (0.10–8.49)	0.56 (0.10–8.49)	1.53 (0.23–7.95)		ns	ns
Initial treatment—*n* (%)								
No treatment		15 (20)	6 (22)	1 (8)	5 (36)		< 0.001	ns
Low efficacy^a^		36 (48)	4 (15)	1 (8)	3 (21)			
Moderate efficacy^a^		12 (16)	1 (4)	1 (8)	0 (0)			
High efficacy^a^		12 (16)	16 (59)	10 (77)	6 (43)			
Escalation of treatment		27 (36)	1 (4)	0 (0)	1 (7)		0.001	ns

Statistical comparisons undertaken were Atypical MS vs Typical MS or Database MS and Tumefactive vs ADEM-like

*MS* multiple sclerosis; *ADEM* acute disseminated encephalomyelitis; *ns* not significant; *CIS* clinically isolated syndrome; *EDSS* expanded disability status scale; *N/A* not applicable

^a^See text for definitions of efficacy

Regression analysis of baseline data on outcome (final MSSS) showed that both male sex (β 2.087 [95% CI 0.981–3.193] and a higher total number of FLAIR T2 hyperintense lesions on MRI brain (β 0.035 [0.012–0.058]) were associated with a worse outcome (Supplementary Table 2 and Supplementary Fig. 2), although the effect of FLAIR T2 lesions was small (*R*^2^ = 0.047). Initial treatment did not significantly affect final MSSS within the atypical MS cohort but was associated with MSSS outcomes (Supplementary Fig. 3) across the whole cohort (*p* = 0.016), with more efficacious therapies being associated with worse outcomes for final MSSS, but not ARR.

### Survival analysis

**Fig. 3:**
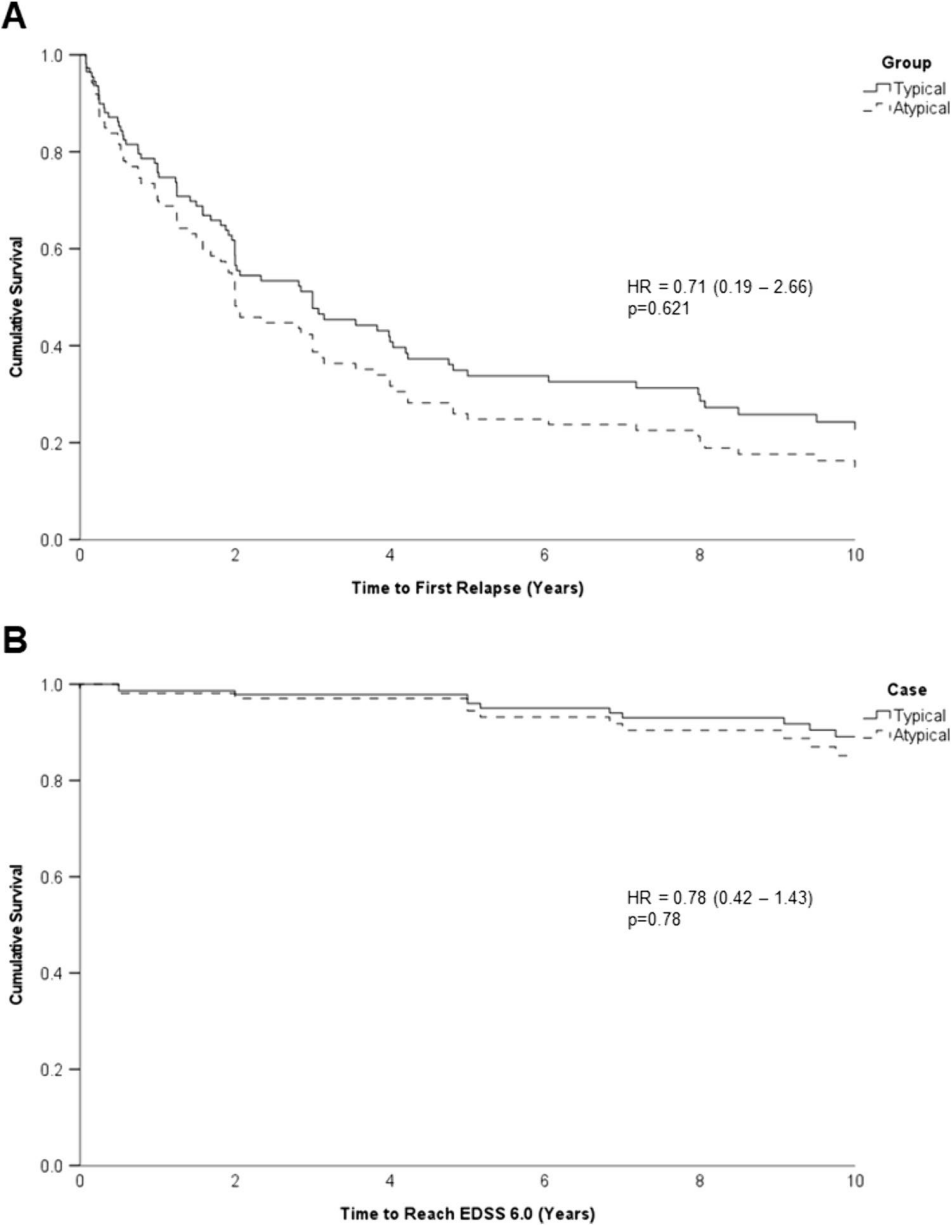
Survival curves from cox-proportional hazard models for time to first relapse (**A**) and time to reach EDSS 6.0 (**B**). Age, sex and initial treatment (low, medium or high efficacy) were included in the model. *EDSS* expanded disability status scale

Cox proportional hazards survival analysis was used for both time to first relapse and time to EDSS 6.0 from first attack (Fig. 3). Only age at onset proved to be statistically significant in this analysis for time to EDSS 6.0 using a forward stepwise approach. However, because of the baseline regression analysis, age, sex and initial treatment were included in the models for both analyses (Supplementary Tables 3 and 4). There were no significant differences in these outcome measures for atypical MS cases compared to typical MS cases. A subgroup analysis looking at ADEM-like and tumefactive cases separately in a Kaplan–Meier analysis (Supplementary Fig. 4) similarly showed no differences in outcomes.

### MRI analysis

**Fig. 4:**
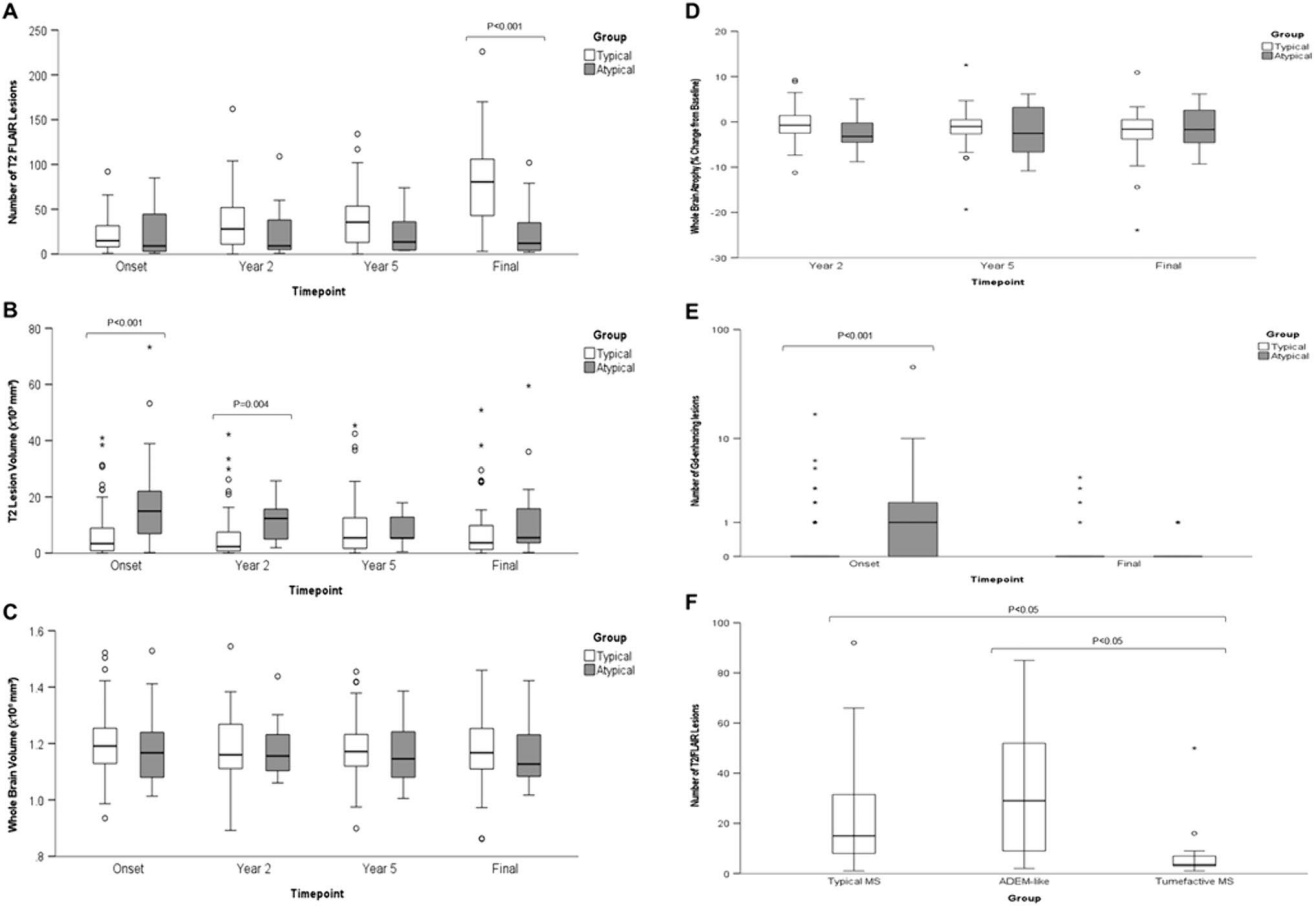
Box and whisker plots of number of T2/FLAIR lesions (**A**), T2 lesion volume (**B**) and whole brain volume (**C**) at presentation of atypical attack (Onset), 2 years, 5 years and last MRI (Final), change in whole brain volume (brain atrophy) compared to baseline at 2 year, 5 year and final follow up (**D**), Number of Gadolinium enhancing lesions at onset and final follow up (**E**), and number of T2/FLAIR lesions at onset for MS, ADEM-like and tumefactive cases (**F**). Significant differences between atypical and typical MS cases are indicated. Central bar shows median, box shows interquartile range and whiskers indicate range. Outliers indicated by circles; extreme outliers indicated by asterisks

**Fig. 5:**
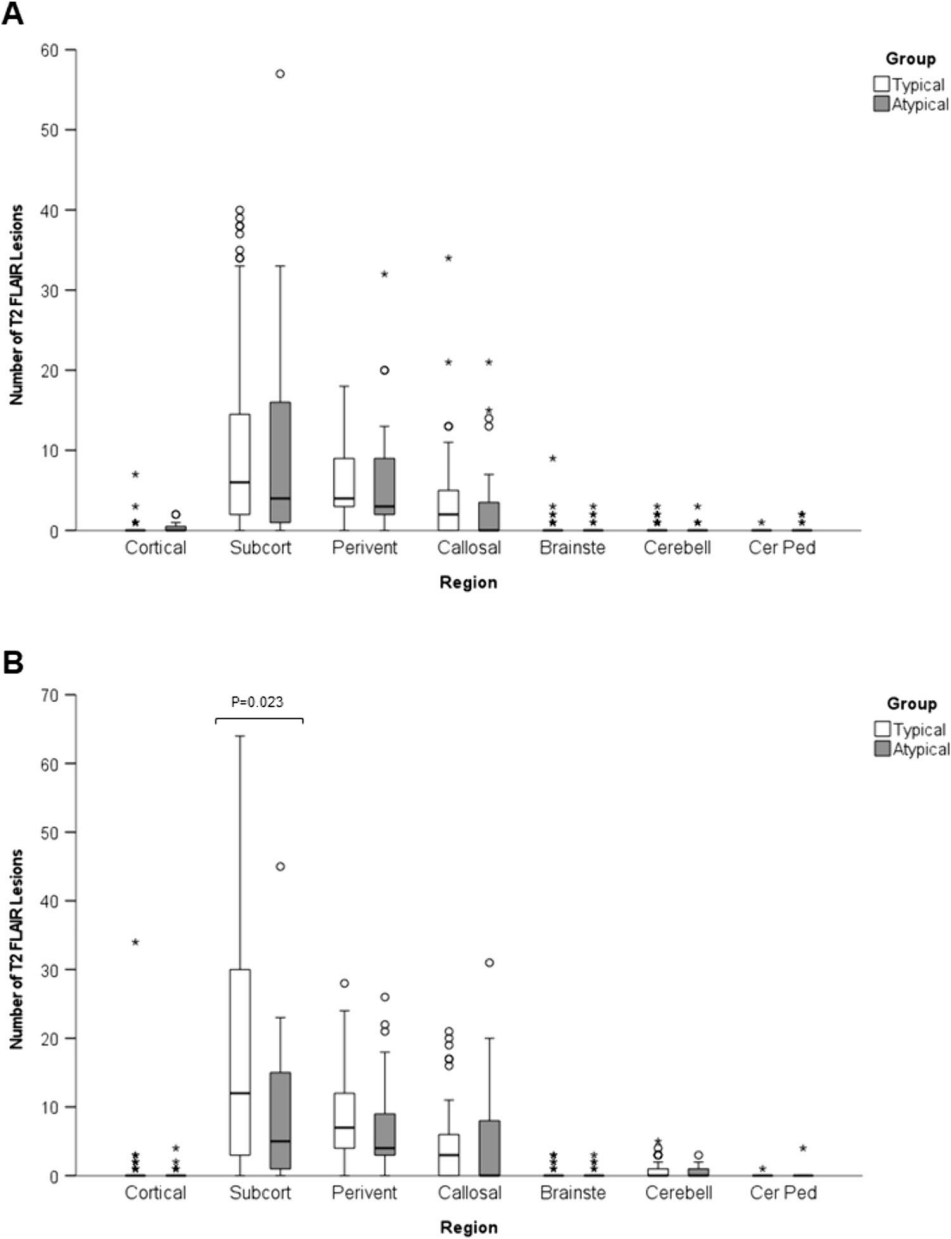
Box and whisker plots of distribution of T2/FLAIR lesions at presentation (**A**) and last follow up (**B**). Significant differences between atypical and typical MS cases are indicated. Central bar shows median, box shows interquartile range and whiskers indicate range. Outliers indicated by circles; extreme outliers indicated by asterisks. *Subcort* subcortical; *Perivent* periventricular; *Brainste* brainstem; *Cerebell* Cerebellar; *Cer Ped* cerebellar peduncle

Analyses of MRI brain with lesion counts, lesion volume and whole brain volume are shown in Fig. 4A–C and Supplementary Table 4. This analysis indicates no significant difference in the number of T2 lesions for typical and atypical cases. More Gd-enhancing lesions were seen in the atypical cases than typical cases (*p* < 0.001) at presentation (Supplementary Table 5. More lesions were evident for the last available MRI in typical MS cases (*p* < 0.001), but this likely reflects the longer period of follow up. T2 lesion volume for atypical cases was higher at disease onset (*p* < 0.001) and at Year 2 (0 = 0.004). However, subsequently there was no significant difference suggesting possible regression to the mean and similar final outcomes. There were no significant differences in whole brain volume or percentage change from baseline in whole brain volume at any timepoint (Fig. 4D and Supplementary Table 4). The number of Gd-enhancing lesions was greater (*p* < 0.001) in the atypical cases than typical MS cases at presentation (Fig. 4E and Supplementary Table 4). As expected, there were fewer T2/FLAIR lesions at presentation in the tumefactive MS group (Fig. 4F). There were more FLAIR lesions at onset in the ADEM-like group compared to typical MS but this difference was not statistically significant. There were no differences in the number of large T2 lesions (> 6 mm) and T1 hypointense lesions (‘old black holes’) at onset or final MRI (Supplementary Fig. 5and Supplementary Table 5). The anatomical distribution of T2 brain lesions showed no significant difference at disease onset (Fig. 5A). Subcortical lesions were more frequent in the typical MS cases at final follow up (Fig. 5B), but this perhaps reflects the greater duration of follow and age of this group. There were no statistically significant differences in the frequency of different lesion features between ADEM-like and tumefactive presentations.

## Discussion

The identified cohort of 28 MS cases with atypical presentations represents approximately 3.9% (95%CI 2.6–5.6%) of cases under the care of GCUH. This prevalence of atypical MS in adults is similar to prior studies (1–5%) [12, 13, 18, 19]. A disproportionately high number of atypical presentations were disproportionally first events (75%) versus what would be expected by chance (34%) amongst those with a relapsing course (*p* < 0.01). This tendency has been noted for tumefactive MS [25], but we observed this pattern in both ADEM-like and tumefactive MS. Recurrent tumefactive lesions were seen in one case, a phenotype that has been previously noted [26]. Frequency of preceding infective symptoms was higher in the atypical group, but this difference was not significant. Previous studies have noted the prevalence of prior infective symptoms, but these prior investigations had no comparison group [13, 27–31]. Atypical clinical features were seen in less than half of the atypical MS cases. The most common presenting symptom in both typical (38%) and atypical (48%) cohorts was sensory deficit, contrasting with previous studies in which motor deficits were the most common presentation in tumefactive MS and ADEM-like presentations [8, 32]. In keeping with previous investigations, multifocal presentations were more common in atypical (56%) compared to typical (5%) MS cases (*p* < 0.001) [13, 29, 32, 33].

AQP-4 antibodies were not detected in our cohort. One ADEM-like case tested positive for MOG antibodies, out of the 21 cases available anti-MOG tested (5%). We acknowledge that this is an incomplete serological data set. Unfortunately, this deficit could not be rectified some of patients were lost to follow-up prior to MOG antibody testing being available. This finding is consistent with prior studies indicating low seroprevalence of AQP4 and anti-MOG antibodies in tumefactive and ADEM-like MS in adults [27, 30, 34–36]. One study suggested a higher prevalence (36%) of AQP4 antibodies in adult tumefactive MS [33]. The low frequency of antibodies contrasts with paediatric cases of ADEM, where MOG antibodies are found to be present in approximately one-half of cases [37]. We acknowledge the controversy of diagnosing a MOG-positive case as atypical multiple sclerosis The primary rationale for inclusion is the subsequent clinical course and radiological progression was more in line with MS than MOG. Potential explanations for this clinical course include treated MOGAD, MS with a false positive MOG antibody or co-incident MOGAD and MS.

We found that, compared to age- and sex-matched typical MS controls, and correcting for follow-up duration, atypical MS showed no difference in long-term outcomes (ARR, MSSS, time to first relapse, time to EDSS 6.0, and number of T2 lesions, T2 lesion volume and brain atrophy at 5 years). This contrasts with some differences seen with the unmatched cohort and measures that were not duration of follow-up adjusted (e.g. final EDSS). This highlights the importance of identifying suitable controls and adjusting for duration of follow up in such studies.

Atypical MS cases were more often commenced on high-efficacy therapy. This likely reflects prognostic concerns in the face of alarming radiological and clinical changes. Interestingly, initial treatment choice did not influence the survival analyses. However, the possibility that differences in initial efficacy of treatment choice may have mitigated natural history differences in long-term outcomes for the two forms of MS needs to be considered [38]. As seen in the majority of prior studies of MS, male sex was associated with greater likelihood of reaching EDSS 6.0 sooner. With a median follow up of 6 years we observed in patients with atypical MS, a conversion to MS on clinical grounds in 17/27 (63%), by MRI criteria in 18/27 (67%) and by both clinical and MRI criteria in 23/27 (85%).

Higher lesion burden within the first 5 years of diagnosis is recognised as conferring increased risk of more severe long term disability in MS [39]. We observed higher T2 lesion volume at disease onset and year 2 in the atypical MS cases (Fig. 4). By year 5 there were no differences and whilst the effect of the number of T2/FLAIR lesions at onset on the time to reach EDSS 6.0 was significant the effect size was small. More lesions were Gd-enhancing at first atypical presentation than in typical MS cases consistent with the florid acute presentations that are commonly seen.

The strengths of this study were that cases were ascertained through a systematically collected single centre database of demyelinating disease cases and comparisons were made with age- and sex-matched typical MS cases selected at random from the same database. In addition, data for typical and atypical cases were collected in the same manner and time-factored outcome measures have been utilised. The weaknesses of this study were that it was retrospective and that whilst matched for age at onset there was a significant difference in the duration of follow up. The inclusion of cases that pre-dated the routine use of volumetric MRI sequences necessitated the use of a less reliable tool for measuring brain volumes. The lack of histopathological correlation is another limitation. However, brain biopsy for evaluation of cerebral lesions has become increasingly rare in clinical practice, given the inherit high risk of complication and the potential of use of imaging characteristics and paraclinical information to identify likely demyelinating lesions pre-biopsy. Furthermore, given the length of follow-up for atypical cases, the presence of alternative diagnoses such as cerebral malignancy would have declared itself clinically or radiologically.

ADEM-like and tumefactive presentations of MS are uncommon. Comparison of clinical features and outcomes with a cohort of typical MS suggests that despite the initial severity of neuro-inflammatory changes, atypical MS presentations result in similar clinical and radiological outcomes (including brain atrophy) to the wider MS population.

### Supplementary Information

Below is the link to the electronic supplementary material.

Supplementary file1 (PPTX 169 KB)

Supplementary file2 (DOCX 14 KB)

Supplementary file3 (DOCX 30 KB)

## Abbreviations

ADEM: Acute disseminated encephalomyelitis
AQP4: Aquaporin-4
CNS: Central nervous system
CSF: Cerebrospinal fluid
DICOM: Digital imaging and communications in medicine
EDSS: Expanded disability severity score
FLAIR: Fluid-attenuated inversion and recovery
GCUH: Gold Coast University Hospital
MOG: Myelin oligodendrocyte glycoprotein
MRI: Magnetic resonance imaging
MS: Multiple Sclerosis
pwMS: Persons with multiple sclerosis
MSSS: Multiple sclerosis severity score
NMOSD: Neuromyelitis optica spectrum disorder
