# Supplementary figures and images for: Long-term outcomes of ADEM-like and tumefactive presentations of CNS demyelination: a case-comparison analysis
Source: J Neurol. 2024 Jun 11;271(8):5275–89. doi: 10.1007/s00415-024-12349-6 (PMC11319424; doi:10.1007/s00415-024-12349-6)

## Slide 1
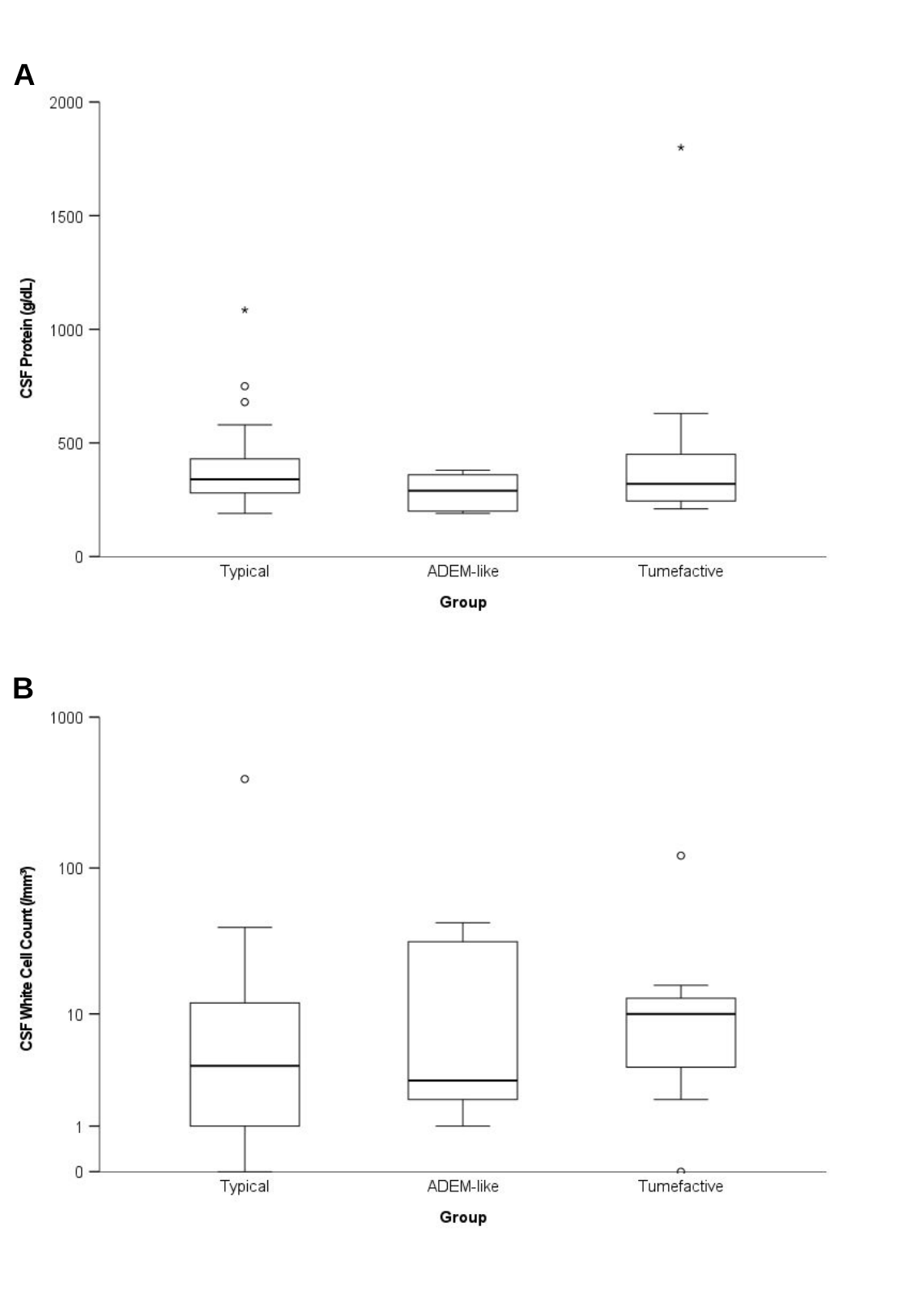

A
B

## Slide 2
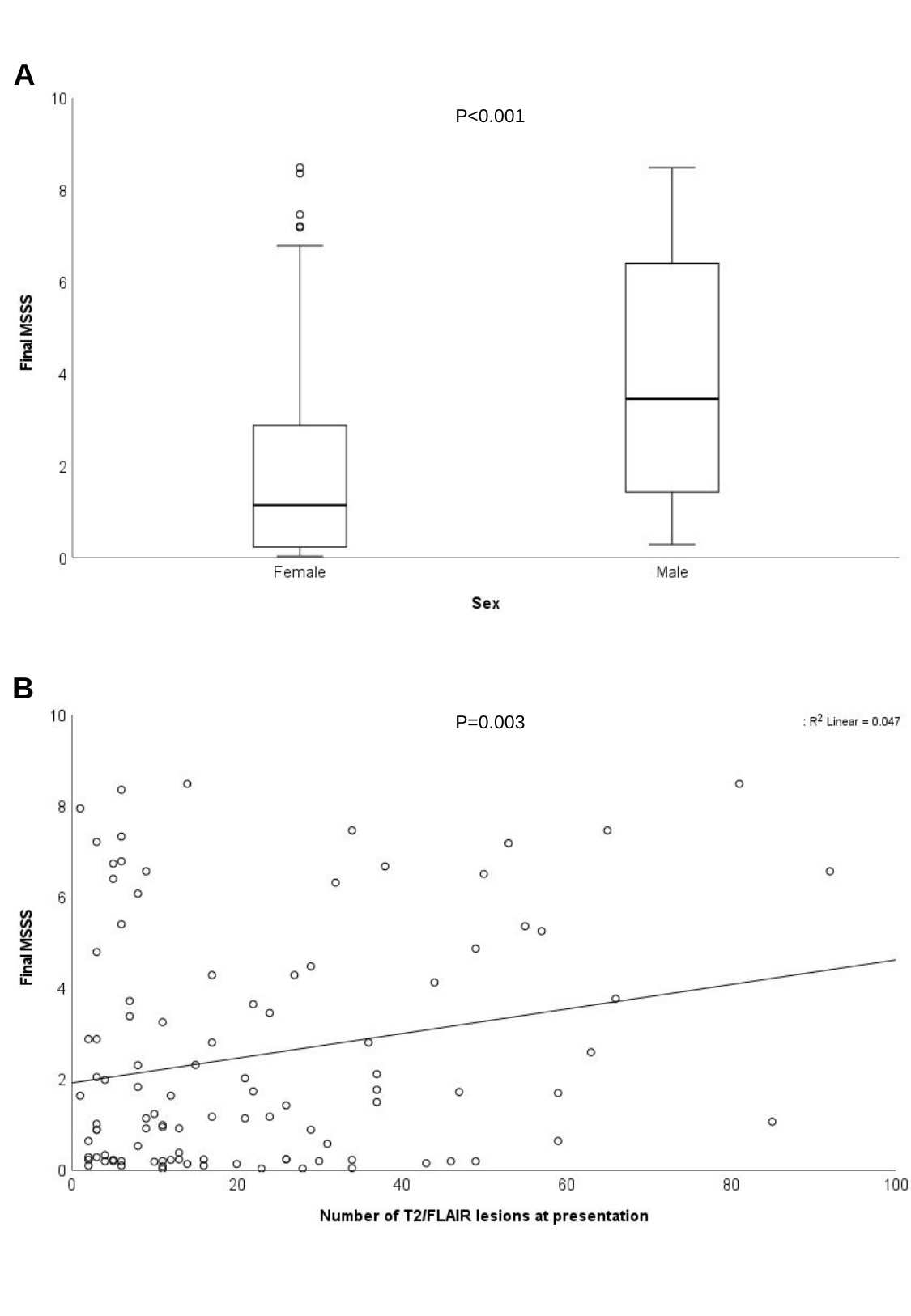

A
P<0.001
B
P=0.003

## Slide 3
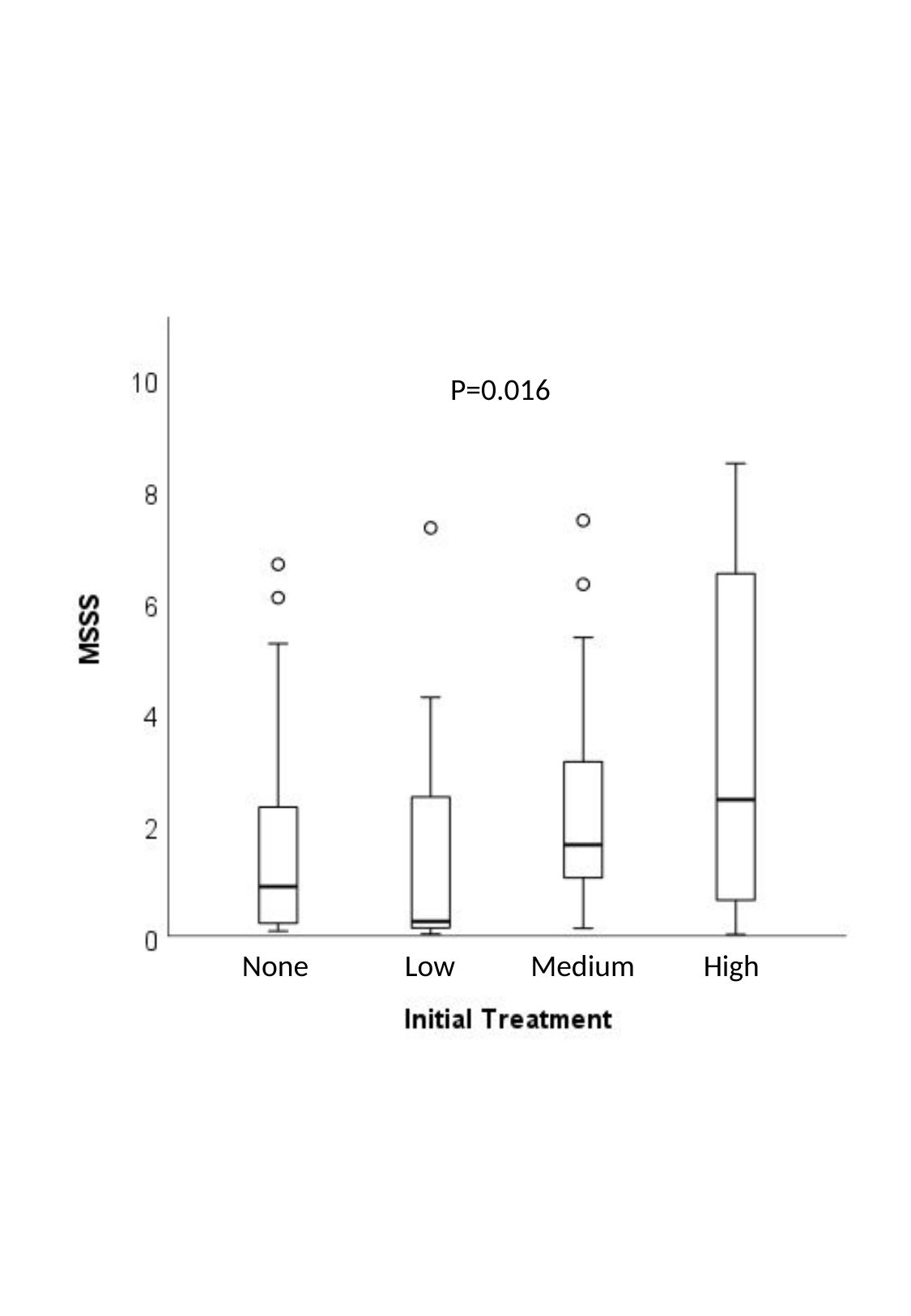

P=0.016
None Low Medium High

## Slide 4
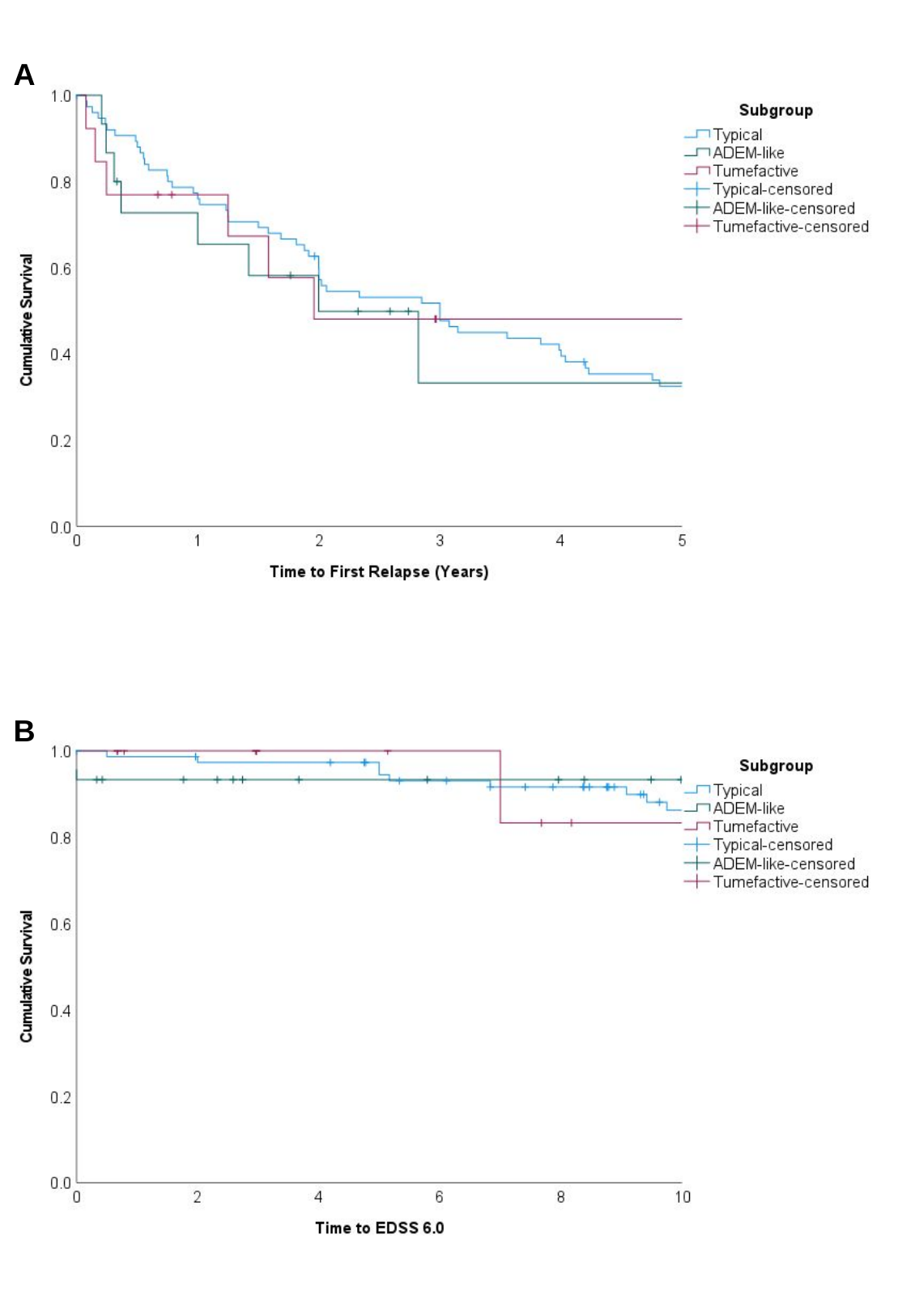

A
B

## Slide 5
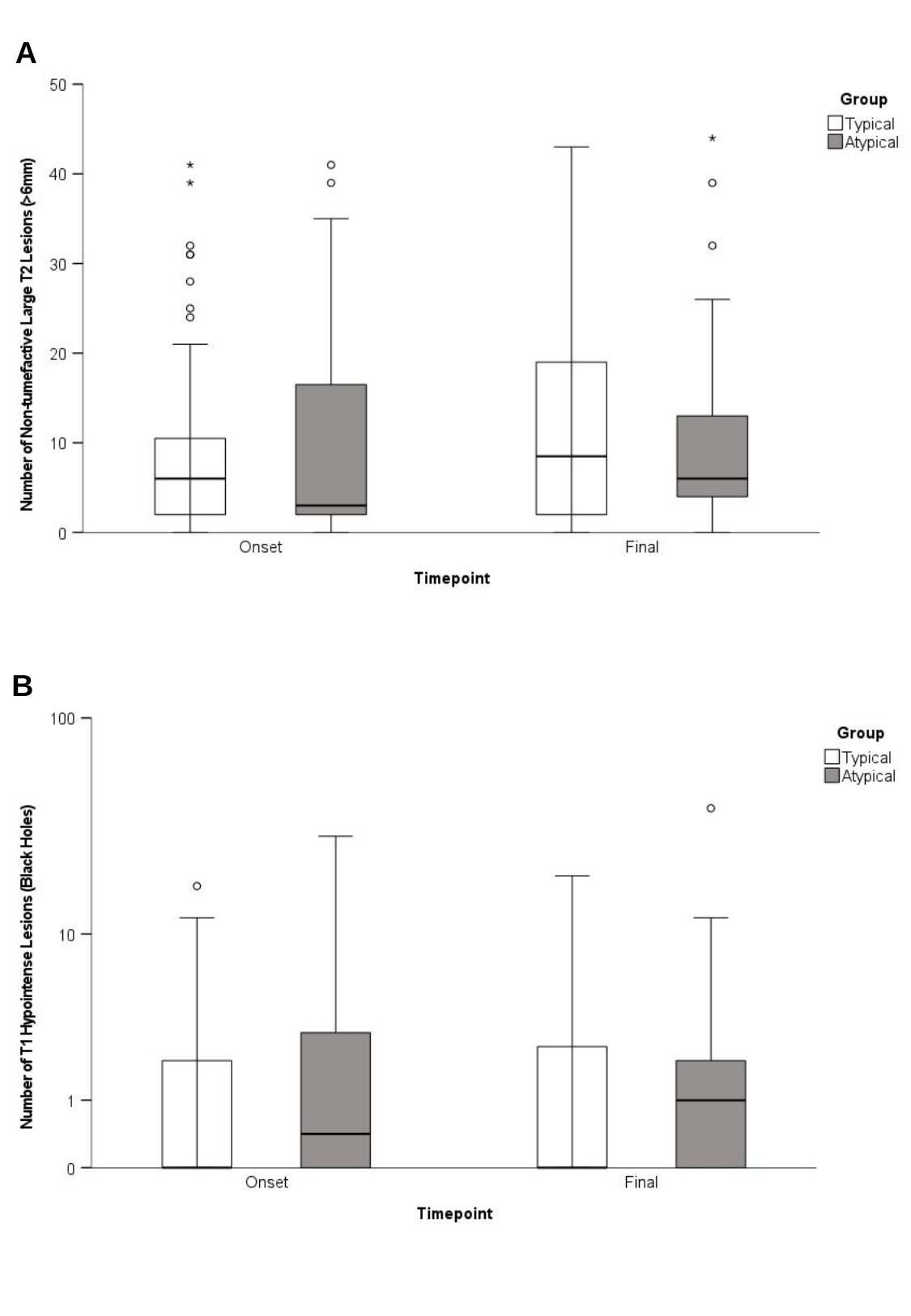

A
B

Supplement: Supplementary file 1 — Supplementary file1 (PPTX 169 KB) [file 415_2024_12349_MOESM1_ESM.pptx]
