# Supplementary material for: Long-term outcomes of ADEM-like and tumefactive presentations of CNS demyelination: a case-comparison analysis
Source: J Neurol. 2024 Jun 11;271(8):5275–89. doi: 10.1007/s00415-024-12349-6 (PMC11319424; doi:10.1007/s00415-024-12349-6)
Supplement: Supplementary file 2 — Supplementary file2 (DOCX 14 KB) [file 415_2024_12349_MOESM2_ESM.docx]

**Supplementary Figure Legends**

**Supplementary Figure 1**

Box and whisker plots of CSF protein levels (A) and CSF white cell count (B – plotted with logarithmic scale) for atypical and typical MS cases. There were no significant differences between atypical and typical MS cases. Central bar shows median, box shows interquartile range and whiskers indicate range. Outliers indicated by circles, extreme outliers indicated by asterisks.

CSF = cerebrospinal fluid; ADEM = acute disseminated encephalomyelitis

**Supplementary Figure 2**

Box and whisker plot of MSSS at last follow up for female and male cases (A). There was no statistical difference between females and males. Central bar shows median, box shows interquartile range and whiskers indicate range. Outliers indicated by circles, extreme outliers indicated by asterisks. Scatter plot of MSSS at final follow up against number of T2/FLAIR brain lesions at presentation. There was no statistically significant relationship between MSSS and T2 lesion count at onset.

MSSS = multiple sclerosis status scale

**Supplementary Figure 3**

Box and whisker plot of final MSSS according to initial treatment efficacy (see text for definitions). Central bar shows median, box shows interquartile range and whiskers indicate range. Outliers indicated by circles, extreme outliers indicated by asterisks.

**Supplementary Figure 4**

Survival curves (Kaplan-Meier) for time to first relapse (A) and time to reach EDSS 6.0 for typical, ADEM-like and tumefactive MS cases. There were statistically significant differences between the three groups for either outcome.

**Supplementary Figure 5**

Box and whisker plots of numbers of non-tumefactive large T2 lesions (>6mm) (A) and hypointense T1 lesions or black holes (B – plotted on logarithmic scale) for presentation and final MRI. There were no significant differences between atypical and typical MS cases at any time point. Central bar shows median, box shows interquartile range and whiskers indicate range. Outliers indicated by circles, extreme outliers indicated by asterisks.
