# Supplementary material for: Long-term outcomes of ADEM-like and tumefactive presentations of CNS demyelination: a case-comparison analysis
Source: J Neurol. 2024 Jun 11;271(8):5275–89. doi: 10.1007/s00415-024-12349-6 (PMC11319424; doi:10.1007/s00415-024-12349-6)
Supplement: Supplementary file 3 — Supplementary file3 (DOCX 30 KB) [file 415_2024_12349_MOESM3_ESM.docx]

**Supplementary Table 1. Summary of MRI lesion features in atypical cases.**

| Case | Type | Multiple tumefactive lesions | Mass effect | Small peri-lesional oedema | Large peri-lesional oedema | Peri-lesional T2 hypointensity | Intralesional T1 hypointensity | DWI Hyperintensity | Any Gd-enhancement | Open/broken ring Gd-enhancement | Closed/complete ring Gd-enhancement | Any central Gd-enhancement | Heterogeneous cenral Gd-enhancement | Homogeneous central Gd-enhancement | Concentric Gd-enhancement |
| --- | --- | --- | --- | --- | --- | --- | --- | --- | --- | --- | --- | --- | --- | --- | --- |
| 1 | ADEM-like | - | - | - | - | - | + | + | - |  |  |  |  |  |  |
| 2 | ADEM-like | + | + | + | + | + | + | + | + | + | + | + | + | + | - |
| 3 | ADEM-like | + | + | + | + | + | + | + | + | + | + | - | - | - | - |
| 4 | ADEM-like | - | - | - | - | - | + | + | + | - | - | + | - | + | - |
| 5 | ADEM-like | - | - | + | - | - | + | - | + | - | - | + | - | + | - |
| 6 | ADEM-like | + | - | + | + | - | + | + | + | + | + | - | - | - | - |
| 7 | ADEM-like | + | - | + | - | - | + | + | + | + | + | + | - | + | - |
| 8 | ADEM-like | - | - | + | - | - | + | + | + | - | - | + | + | - | - |
| 9 | ADEM-like | + | + | + | + | - | + | - | - |  |  |  |  |  |  |
| 10 | ADEM-like | - | + | + | + | - | + | + | - |  |  |  |  |  |  |
| 11 | ADEM-like | + | - | - | - | - | + | + | - |  |  |  |  |  |  |
| 12 | ADEM-like | - | - | - | - | - | + | + | - |  |  |  |  |  |  |
| 13 | ADEM-like | - | + | + | + | - | - | + | - |  |  |  |  |  |  |
| 14 | Tumefactive | + | - | + | - | - | + | + | + | - | - | + | + | - | - |
| 15 | Tumefactive | - | - | + | - | - | + | + | + | - | - | + | - | - | - |
| 16 | Tumefactive | + | - | + | - | - | + | + | + | - | + | - | - | - | - |
| 17 | Tumefactive | + | - | + | - | - | - | + | + | - | - | + | + | - | - |
| 18 | Tumefactive | + | - | + | - | + | + | + | + | - | - | + | + | - | - |
| 19 | Tumefactive | + | + | - | + | - | + | + | + | + | - | - | - | - | - |
| 20 | Tumefactive | + | - | - | + | + | + | + | + | - | - | + | + | + | - |
| 21 | Tumefactive | - | - | - | - | - | + | + | - |  |  |  |  |  |  |
| 22 | Tumefactive | + | - | + | - | - | + | + | + | + | - | - | - | - | - |
| 23 | Tumefactive | + | - | + | - | - | + | + | + | + | - | + | + | - | - |
| 24 | Tumefactive | + | - | + | - | - | + | + | + | - | - | + | + | - | - |
| 25 | Tumefactive | + | - | - | + | + | + | + | + | - | + | + | + | - | - |
| 26 | Tumefactive | - | + | + | - | - | + | + | - |  |  |  |  |  |  |
| 27 | Tumefactive | - | - | - | - | - | - | - | - |  |  |  |  |  |  |

**Supplementary Table 2. Results of baseline predictor regression analysis of final MSSS**

| Variable | β (95% CI) | Std Error | Standardised β | t | P-value |
| --- | --- | --- | --- | --- | --- |
| Sex (Male) | 2.087 (0.981- 3.193) | 0.557 | 0.350 | 3.745 | <0.001 |
| No. FLAIR Lesions | 0.035 (0.012-0.058) | 0.012 | 0.281 | 3.006 | 0.003 |

No. = number

**Supplementary Table 3. Cox proportional hazards model for time to first relapse**

| Clinical Feature | β | Wald | P-value | HR (95% CI) |
| --- | --- | --- | --- | --- |
| Age of onset | -0.100 | 0.492 | 0.483 | 0.990 (0.962 – 1.018) |
| Sex | -0.184 | 0.333 | 0.564 | 0.832 (0.446 – 1.553) |
| Initial treatment | -0.127 | 1.091 | 0.296 | 0.881 (0.694 – 1.118) |

HR = hazard ratio

**Supplementary Table 3. Cox proportional hazards model for time to reach EDSS 6.0**

| Clinical Feature | β | Wald | P-value | HR (95% CI) |
| --- | --- | --- | --- | --- |
| Age of onset | 0.057 | 4.805 | 0.028 | 1.058 (1.006 – 1.114) |
| Sex | 0.670 | 2.142 | 0.143 | 1.954 (0.797 – 4.795) |
| Initial treatment | -0.075 | 0.114 | 0.735 | 0.928 (0.602 – 1.431) |

HR = hazard ratio

**Supplementary Table 4. Raw Data for Figures 4 and 5, and Supplementary Figure 5.**

| MRI Feature | Onset | | | Year 2 | | | Year 5 | | | Final | | |
| --- | --- | --- | --- | --- | --- | --- | --- | --- | --- | --- | --- | --- |
|  | Typical | Atypical | p-value | Typical | Atypical | p-value | Typical | Atypical | p-value | Typical | Atypical | p-value |
| N | 75 | 26 |  | 60 | 13 |  | 59 | 9 |  | 74 | 26 |  |
| Whole Brain Volume (x10^6^ mm^3^) | 1.19 (0.94 - 1.52) | 1.17 (0.10 - 1.53) | ns | 1.16 (0.89 - 1.54) | 1.16 (1.06 - 1.44) | ns | 1.17 (0.90 - 1.46) | 1.15 (1.01 - 1.39) | ns | 1.17 (0.86 - 1.46) | 1.13 (1.02 - 1.42) | ns |
| T2 Lesion Volume (x10^3^ mm^3^) | 3.41 (0.02 - 40.92) | 14.95 (0.20 - 73.28) | <0.001 | 2.31 (0 - 42.19) | 12.33 (1.88 - 25.73) | 0.004 | 5.42 (0.03 - 4.54) | 5.36 (0.45 - 1.79) | ns | 3.70 (0.02 - 5.08) | 5.46 (0.22 - 5.95) | ns |
| Whole Brain Atrophy (% Change from Baseline) |  |  |  | -0.73 (-11.24 - +9.27) | -3.21 (-8.79 - +5.05) | ns | -1.05 (-19.33 - +12.54) | -2.53 (-10.81 - +6.15) | ns | -1.62 (-23.91 - +10.91) | -1.62 (-9.29 - +6.15) | ns |
| Number of T2 FLAIR Lesions | 15 (1 - 92) | 9 (1 - 85) | ns | 28 (0 - 162) | 9 (1 - 109) | ns | 35.5 (0 - 134) | 13.5 (4 - 74) | 0.079 | 80.5 (3 - 226) | 12 (2 - 102) | <0.001 |
| Number of Non-tumefactive Large T2 Lesions (>6mm) | 6 (0 - 41) | 3 (0 - 41) | ns |  |  |  |  |  |  | 8.5 (0 - 43) | 6 (0 - 44) | ns |
| Number of Gd-enhancing T1 Lesions | 0 (0 -17) | 1 (0 - 46) | <0.001 |  |  |  |  |  |  | 0 (0 - 4) | 0 (0 - 1) | ns |
| Number of T1 Hypointense Lesions (Black Holes) | 0 (0 -17) | 0.5 (0 - 29) | ns |  |  |  |  |  |  | 0 (0 - 19) | 1 (0 - 39) | ns |

All statistics reported as median (range)

MRI = magnetic resonance imaging; Gd = gadolinium

**Supplementary Table 5. Additional MRI parameters**

| MRI Feature | Onset | | | Final | | |
| --- | --- | --- | --- | --- | --- | --- |
|  | Typical | Atypical | p-value | Typical | Atypical | p-value |
| N | 75 | 26 |  | 74 | 26 |  |
| Number of Gd-enhancing Lesions | 0 (0 - 17) | 1 (0 - 46) | <0.001 | 0 (0 - 4) | 0 (0 - 1) | ns |
| Number of T1 Hypodense Lesions | 0 (0 -17) | 0.5 (0 - 29) | ns | 0 (0 - 19) | 1 (0 - 39) | ns |
| Number of Large T2 Lesions | 6 (0 - 41) | 3 (0 - 41) | ns | 8.5 (0 - 43) | 6 (0 - 44) | ns |
| Number of Old T1 Hypodense Lesions | 0 (0 - 37) | 0 (0 - 29) | ns |  |  |  |

MRI = magnetic resonance imaging; Gd = gadolinium

**Supplementary Table 6. Number of available MRIs at initial presentation, year two, year five and final review**

| **MRI analysis type** | **Initial MRI** | | **Year 2** | | **Year 5** | | **Final MRI** | |
| --- | --- | --- | --- | --- | --- | --- | --- | --- |
|  | Typical | Atypical | Typical | Atypical | Typical | Atypical | Typical | Atypical |
| Lesion count (n) | 76 | 27 | 55 | 13 | 56 | 8 | 75 | 25 |
| Whole brain and lesion volume (n) | 75 | 25 | 61 | 14 | 59 | 9 | 74 | 25 |

MRI = Magnetic resonance imaging
